# Characterization of *Neochloris oleoabundans* under Different Cultivation Modes and First Results on Bioactivity of Its Extracts against HCoV-229E Virus

**DOI:** 10.3390/plants12010026

**Published:** 2022-12-21

**Authors:** Costanza Baldisserotto, Valentina Gentili, Roberta Rizzo, Chiara Di Donna, Luna Ardondi, Annalisa Maietti, Simonetta Pancaldi

**Affiliations:** 1 Department of Environmental and Prevention Sciences, University of Ferrara, C.so Ercole I d’Este, 32, 44121 Ferrara, Italy; 2 Department of Chemical, Pharmaceutical and Agricultural Sciences, University of Ferrara, Via Luigi Borsari, 46, 44121 Ferrara, Italy

**Keywords:** *Neochloris oleoabundans*, HCoV-229E, microalgae, *Coronaviridae*, antiviral, total extracts

## Abstract

Microalgae are proposed in several biotechnological fields because of their ability to produce biomass enriched in high-value compounds according to cultivation conditions. Regarding the health sector, an emerging area focuses on natural products exploitable against viruses. This work deals with the characterization of the green microalga *Neochloris oleoabundans* cultivated under autotrophic and mixotrophic conditions as a source of whole aqueous extracts, tested as antivirals against HCoV-229E (*Coronaviridae* family). Glucose was employed for mixotrophic cultures. Growth and maximum quantum yield of photosystem II were monitored for both cultivations. Algae extracts for antiviral tests were prepared using cultures harvested at the early stationary phase of growth. Biochemical and morphological analyses of algae indicated a different content of the most important classes of bioactive compounds with antiviral properties (lipids, exo-polysaccharides, and total phenolics, proteins and pigments). To clarify which phase of HCoV-229E infection on MRC-5 fibroblast cells was affected by *N. oleoabundans* extracts, four conditions were tested. Extracts gave excellent results, mainly against the first steps of virus infection. Notwithstanding the biochemical profile of algae/extracts deserves further investigation, the antiviral effect may have been mainly promoted by the combination of proteins/pigments/phenolics for the extract derived from autotrophic cultures and of proteins/acidic exo-polysaccharides/lipids in the case of mixotrophic ones.

## 1. Introduction

Microalgae is a large group of photosynthetic micro-organisms, mostly distributed in fresh and marine waters but also in adverse environments [1,2]. Due to their evolutionary history and their high adaptability to variations in environmental conditions, these micro-organisms have evolved remarkable diversity of physiological and biochemical constraints [3,4,5], making them promising natural sources for a wide range of biotechnological applications (for example, production of food/feed, nutraceuticals, pharmaceuticals, green-energy) [5,6,7,8,9]. Taking this into account, artificial manipulations of growth conditions (nutrients, temperature, salinity, etc.) are often linked to the variation of microalgae metabolism so that these micro-organisms are induced to modify their morphology and/or to synthesize or also accumulate different compounds frequently linked to bioactivity [8,10,11,12,13,14]. Among different cultivation methods, compared to autotrophy, mixotrophy is a challenging strategy for the biotechnological exploitation of microalgae since not only is it associated with variations in the biochemical composition of algal biomass but also with higher productivity [15]. Indeed, in mixotrophic cultivations, organic carbon sources are added to mineral culture media, thus allowing microalgae to perform photosynthesis and respiration of external organic carbon concomitantly; this improves growth but also modifies the overall metabolism, often inducing, for example, enhanced lipid or exo-polysaccharides (EPS) production [12,15,16].

Focusing on the health sector, microalgae are a source of various bioactive compounds, like pigments, vitamins, polyunsaturated fatty acids, phenolics, polysaccharides, or proteins, which have been studied for their antibacterial, antiviral, anti-inflammatory, antioxidant, anticancer and other pharmacological activities [12,17,18]. As concerns microalgae with antiviral properties, promising results come from the cyanobacteria *Spirulina platensis* (also referred to *Arthrospira platensis*) and *Nostoc ellipsosporum*, and from the Chlorophyta *Dunaliella primolecta* or *Chlorella vulgaris* and *C. pyrenoidosa* [19,20,21,22]; little of the literature information is available on other microalgae [19,23,24]. As an example, according to Scopus^®^, around 200 documents with “microalgae” and “antiviral” as keywords were published from 1985 up to 2022; narrowing the search to “Spirulina” or “*Nostoc*” or “*Chlorella*” or “*Dunaliella*” and “antiviral” documents were, respectively, 126, 55, 48 and 19; papers referring to other microalgae were usually below 10 (searched genera: *Scenedesmus*, *Chlamydomonas*, *Neochloris*, *Tetraselmis*, *Amphora*). However, very recently, the green microalga *Neochloris oleoabundans*, mainly studied for its capability to accumulate lipids for the bio-energetic sector [10,25,26,27], has been proposed for some applications in the health sector [12,17,28]. In detail, [12] recently characterized the polysaccharidic profile and production of the alga, and interesting immunomodulatory properties have been highlighted for the first time; [28] evaluated the antiproliferative activity of bioactive carotenoids from *N. oleoabundans* against human colon cancer cells; and [17] proposed aqueous extracts of the alga as a natural-oriented and vegan bioactive ingredient for dermocosmetic purposes thanks to antioxidant properties. Furthermore, it is noteworthy that *N. oleoabundans* can grow mixotrophically and, for the over-production of bioactive compounds, like lipids/fatty acids [10] or EPS [12], the addition of glucose to the culture medium represents an excellent strategy. This makes the alga a new further candidate to be exploited in the biomedical-pharmaceutical field as a source of new natural products with anti-microbial and even antiviral activity.

Viruses are commonly considered non-living entities, being made up of DNA or RNA as a core of genetic material surrounded by a protective protein shell called capsid [29]. Even if they are the smallest entities on Earth, they can cause severe infections both to humans and animals via a pathway that, in summary, involves three steps: 1. Binding of the virus to a cell plasma membrane receptor, with a consequent transfer of the virus genome into the cytoplasm of the cell; 2. Reproduction of the virus genome by the host cell; 3. Production of proteins and virions progeny [30]. Among virus families, *Coronaviridae* includes seven virus species able to infect humans (hCoV-229E, hCoV-NL63, hCoV-OC43, hCoV-HKU1, MERS-CoV, SARS-CoV-1 and SARS-CoV-2), causing respiratory diseases. MERS-CoV, SARS-CoV-1 and SARS-CoV-2 cause highly pathogenic viral respiratory infections: Middle East Respiratory Syndrome, Severe Acute Respiratory Syndrome and COVID-19 disease, respectively. As is well known, the last one caused a pandemic that started in Wuhan (China) in 2019 and then rapidly widespread all over the planet. *Coronaviridae* family groups large, spherical, enveloped, positive single-stranded RNA viruses [31,32]. *Coronaviridae* encodes for several proteins, such as the nucleocapside protein, the spike glycoprotein, which mediates virus entry into the host cells, and the capsidic and envelope proteins, which are involved in the virus assembly [33,34]. In human coronaviruses, the spike protein is involved in the host-virus interactions and has been widely studied in recent years as a target for antiviral studies against SARS-CoV-1 and MERS-CoV [34,35]. In the last three years, due to the highly severe health pathologies associated with SARS-CoV-2, a large effort by the scientific community has been massively driven towards finding effective strategies to counteract it. In addition to synthetic drugs or vaccines already available, an opportunity is given by naturally sourced products, which could prevent complications associated with the use of synthetic drugs. Within this perspective, microalgae can be studied for their possible positive effects also against pathologies linked to *Coronaviridae* infections.

Thus, the present work was aimed at treating HCoV-229E, a not-aggressive model virus useful to approach preliminary studies on *Coronaviridae* viruses, with whole extracts of the green microalga *N. oleoabundans*. The alga was cultivated under autotrophic (A) and mixotrophic (M) conditions, was morpho-physiologically and biochemically evaluated and used to produce whole aqueous extracts for anti-viral tests. In the present work, the alga was characterized to highlight differences potentially underlying the effects of its extracts on the HCoV-229E virus.

## 2. Results

### 2.1. Growth Parameters

**Figure 1:**
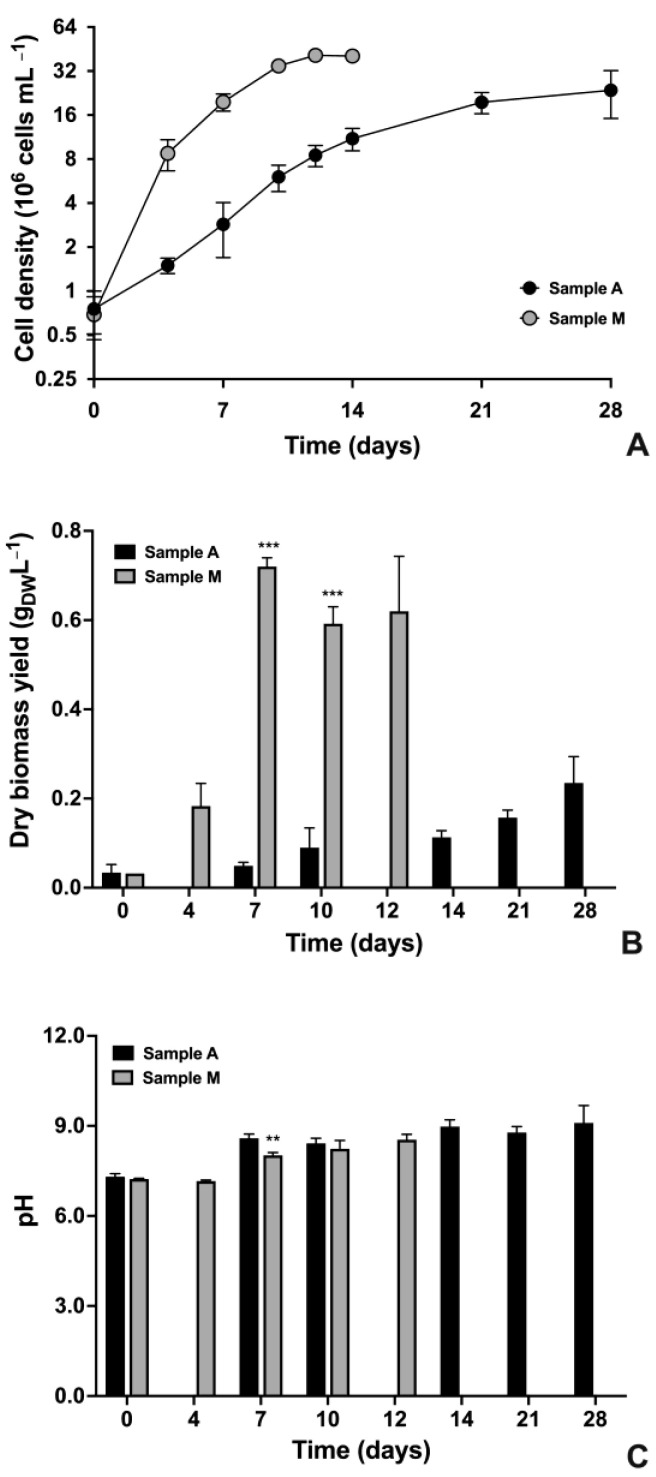
Growth parameters of autotrophic (A) and mixotrophic (M) cultures of *N. oleoabundans* during a cultivation period of 28 d for culture A and of 12 or 14 d for culture M. (**A**) Growth kinetics plotted on a base 2 logarithmic scale; cell density values of cultures were used (10^6^ cells mL^−1^). (**B**) Dry biomass yield (g_DW_ L^−1^). (**C**) pH trend in culture media harvested from samples A and M. In (**A**), sample A, black circles; sample M, grey circles. In (**B**,**C**), sample A, black histograms; sample M, grey histograms. Data refer to means ± standard deviations (*n* ≥ 3). In (**A**,**B**), differences between samples A and M were always significant (*p* < 0.001; ***), except at the inoculation time (time 0). In (**C**), differences between samples A and M were significant (*p* < 0.01; **) only at day seven.

By considering growth parameters, it immediately emerges that different results characterized autotrophic and mixotrophic cultures, respectively named A and M (Figure 1). Even if starting from similar values of cell density and dry biomass, the two cultures underwent different growth kinetics (Figure 1A). Culture M reached the highest values of cell density already at 12–14 d of cultivation with about 40 × 10^6^ cells mL^−1^, while culture A never reached those values and obtained its highest concentration (around 22 × 10^6^ cells mL^−1^) only at the end of the cultivation period (28 d) (Figure 1A) (*p* < 0.01 by comparing data at the end of cultivation). In particular, culture A showed a 10 d-long exponential phase of growth, followed by a prolonged late exponential phase during the time interval 10–21 d. At 28 d, the stationary phase was reached. Differently, culture M immediately entered an intense exponential phase of growth, which lasted from 0 to 4 d of cultivation, and was followed by a 6 d-long late exponential phase (time interval 4–10 d of cultivation). For culture M, the stationary phase was reached in only 12 d, and cell density started to decrease already at 14 d (Figure 1A). Linked to the differences mentioned above and as can be clearly seen from the different slopes of the growth curves during the exponential phase (Figure 1A), culture A had a growth rate of about 0.39 d^−1^, while culture M of even 0.94 d^−1^, i.e., about 2.4 times higher than that obtained for culture A (*p* < 0.001).

Parallel to cell density, at the main cultivation times (0, 7, 10, 14, 21 and 28 d for culture A; 0, 4, 7, 10, 12 d for culture M), dry biomass yield and pH were also evaluated (Figure 1B,C). For both cultures, dry biomass increased quite following the trend recorded for cell densities; in culture A, it reached the highest value at the end of cultivation (0.235 g_DW_ L^−1^, 28 d), while in culture M the highest dry biomass yield was obtained at day 7 (0.720 g_DW_ L^−1^), and then settled at slightly lower values of about 0.62 g_DW_ L^−1^ at the end of cultivation (day 12), namely 2.65 times higher than the highest value recorded for culture A (Figure 1B). As regards the pH of cultures, starting from values ranging around 7.2–7.3 at the inoculation time, in culture A, it quickly increased to values over 8.5 during the first week of cultivation; then it continued to increase a little to 9.10 at 28 d (Figure 1C) (*p* < 0.001 by comparing data at t0 with those from t7 to t28). Differently, in culture M, pH remained substantially unchanged in the time interval 0–4 d (*p* > 0.05), but then it strongly increased to values of about 8 from day four to day seven of cultivation, and subsequently, it stabilized at 8.2–8.5 at 10–12 d of cultivation (Figure 1C) (*p* < 0.001 by comparing data at t0 with those from t7 to t12).

### 2.2. Photosynthetic Parameters and Chemical Composition of Algae

#### 2.2.1. PSII Maximum Quantum Yield and Photosynthetic Pigment Content

**Figure 2:**
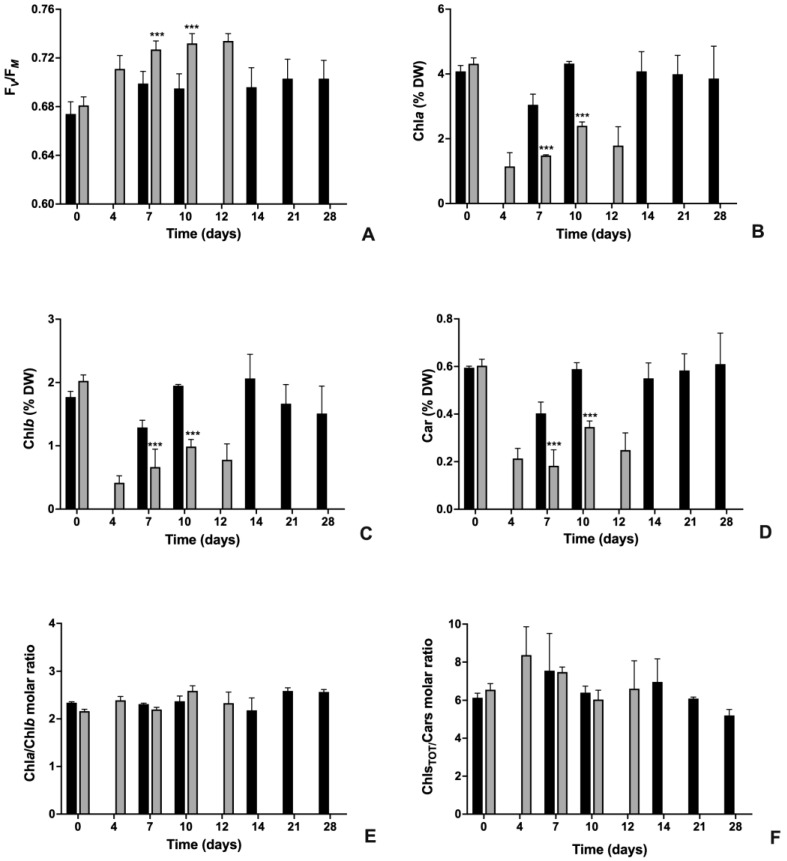
Time-course variations of the photosynthetic parameters of autotrophic (A) and mixotrophic (M) cultures of *N. oleoabundans* during a cultivation period of 28 d and 12 d for culture A and culture M, respectively. (**A**) Maximum quantum yield of PSII (F_V_/F_M_ ratio). (**B**) Chlorophyll *a* content (Chl*a*, %DW). (**C**) Chlorophyll *b* content (Chl*b*, %DW). (**D**) Carotenoids content (Car, %DW). (**E**) Chlorophyll *a* over chlorophyll *b* molar ratio (Chl*a*/Chl*b*). (**F**) Total chlorophylls over carotenoids molar ratio (Chls_TOT_/Cars). In (**E**,**F**), molar concentration of pigments (nmol_PIG_ × 10^−6^ cells) was used for calculations. Black histograms, culture A; grey histograms, culture M. Data refer to means ± standard deviations (*n* = 3). When present, at each cultivation time, asterisks identify significant differences between cultures A and M as follows: ***, *p* < 0.001.

In order to evaluate if cultures A and M showed differences at the photosynthetic metabolism level both in terms of physiological aspects and chemical composition, variations of PSII maximum quantum yield and the content of the photosynthetic pigment were assessed (Figure 2).

As reported in Figure 2A, at the inoculation time (t0), both cultures were characterized by similar F_V_/F_M_ values, ranging around 0.680 (*p* > 0.05). In both cultures, the maximum efficiency of PSII photochemistry significantly increased during cultivation but with evident differences in trend and values. For culture A, in a week of cultivation, F_V_/F_M_ increased to values of about 0.700, which remained substantially unchanged up to the end of the cultivation period (0.703 at 28 d) (*p* < 0.001 with respect to t0). Differently, for culture M, values of PSII maximum quantum yield have risen even more; in detail, at day 4, F_V_/F_M_ already gave values of about 0.710, but it continued to increase up to 0.734 at day 12 (Figure 2A) (*p* < 0.001 with respect to t0). At all experimental times, except t0, F_V_/F_M_ values recorded for culture M were significantly higher than those obtained from culture A (*p* < 0.001).

As regards the content of the photosynthetic pigment (Figure 2B–D), the two algae cultures started with similar values at time 0 (*p* > 0.05 in all cases); thereafter, in culture M, concentrations of pigments were always significantly lower than those in culture A (*p* < 0.001). In detail, in algae belonging to culture A, Chl*a*, Chl*b* and carotenoids tended to maintain almost stable values of about 4, 1.5–2 and 0.6 %DW, respectively (Figure 2B–D). Only at seven days of cultivation the concentration of all pigments was significantly lower with respect to that measured at time 0 and at the other experimental times (Anova; *p* < 0.05). Differently, in culture M algae, the concentration of all pigments tended to strongly decrease especially during the first four to seven days of cultivation: in detail, on both day four and day seven, all pigments were about only 20–30% with respect to the concentrations recorded at the inoculation time (Anova; *p* < 0.05). During the subsequent period of cultivation, concentrations tended to increase, even if they never reached initial values. Regardless of the variations recorded in the concentration of the individual pigments, the Chl*a* over Chl*b* molar ratio ranged around 2.2–2.6 for both cultures, while the total Chls over Cars molar ratio around 6.4 for culture A (maximum–minimum values: 7.56–5.2 at day 7 and 28, respectively) and 7 for culture M (maximum–minimum values: 8.34–6.04 at day 4 and 10, respectively) (Figure 2E,F).

#### 2.2.2. Quantification of Total Proteins and Total Phenolics

Total proteins and total phenolic compounds were quantified on algae harvested at time 28 and 12 d, respectively, for cultures A and M, i.e., when the best compromise among results in terms of growth, physiological status (F_V_/F_M_) and photosynthetic pigment concentration of cultures were obtained (Figure 1 and Figure 2).

**Figure 3:**
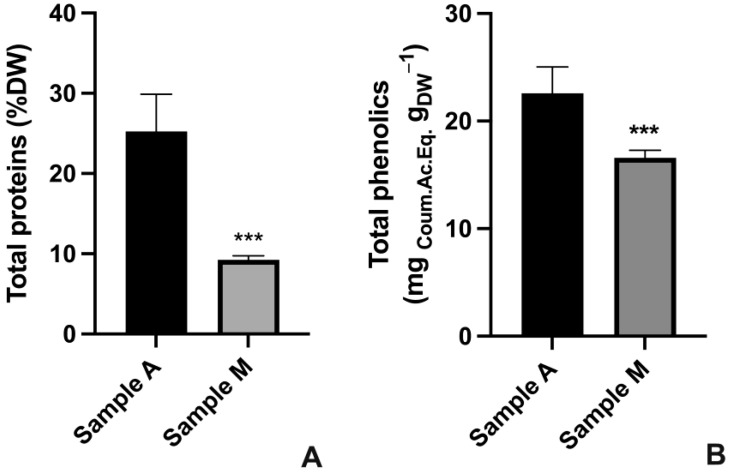
Total protein and total phenolic compounds content in *N. oleoabundans* autotrophic (A) and mixotrophic (M) cultures at 28 d for culture A and at 12 d for culture M. (**A**) Total protein content expressed as percentage of algal dry biomass (%DW). (**B**) Total phenolic compounds content expressed as mg equivalents of coumaric acid over algal biomass (mg_Coum.Ac.Eq._ g_DW_^−1^). Data refer to means ± standard deviations (*n* = 3). Asterisks identify significant differences between cultures A and M: ***, *p* < 0.001.

As reported in Figure 3, comparing the two algae cultures, the highest content of both total proteins and total phenolics was recorded in culture A (about 3× and 1.36× for proteins and phenolics, respectively). In detail, total proteins were about 25 %DW in culture A cells and only 9 %DW in culture M cells (Figure 3A), while total phenolic compounds were about 22.6 and 16.6 mg_Coum.Ac.Eq_ g_DW_^−1^ for samples A and M, respectively (Figure 3B).

### 2.3. Observations of Algae Cell Morphology

**Figure 4:**
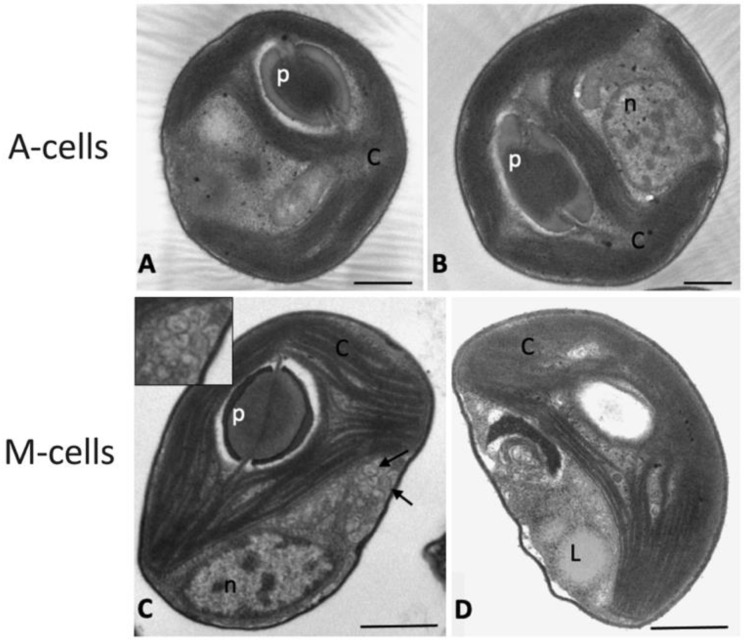
Transmission electron microscopy images of *N. oleoabundans* cells belonging to autotrophic (**A**,**B**) and mixotrophic (**C**,**D**) cultures at 28 and 12 d of cultivation, respectively. A-cells refer to algae in autotrophic cultures, while M-cells refer to algae in mixotrophic ones. C, chloroplast; p, pyrenoid; n, nucleus; L, lipid globule; arrows, cytoplasmic vesicles. In (**C**), insert shows a detail of vesicles at larger magnification. Bars: 0.5 μm.

The ultrastructure of algae belonging to samples A and M was intended to highlight the overall feature of algae and the possible presence or accumulation of compounds of biotechnological interest (Figure 4). In sample A, cells had an almost spherical shape, with a cell diameter of about 2.5–3 µm and a well-structured nucleus (Figure 4A,B). Most of the cell volume was occupied by a large cup-shaped chloroplast with elongated and appressed thylakoid membranes and an evident pyrenoid, surrounded by a starch shell and crossed by one-two thylakoids (Figure 4A,B). Different from cells in sample A, M cells were slightly smaller and flattened in shape (about 2.2 × 1.5 µm, major × minor axis), but, similar to A cells, they contained a large chloroplast with appressed thylakoid membranes and an evident pyrenoid (Figure 4C,D). In some cases, cells showed the formation of vesiculations in the area next to the plasma membrane (Figure 4C), or cytoplasmic not-electron-dense globules, due to lipid accumulation (Figure 4D).

**Figure 5:**
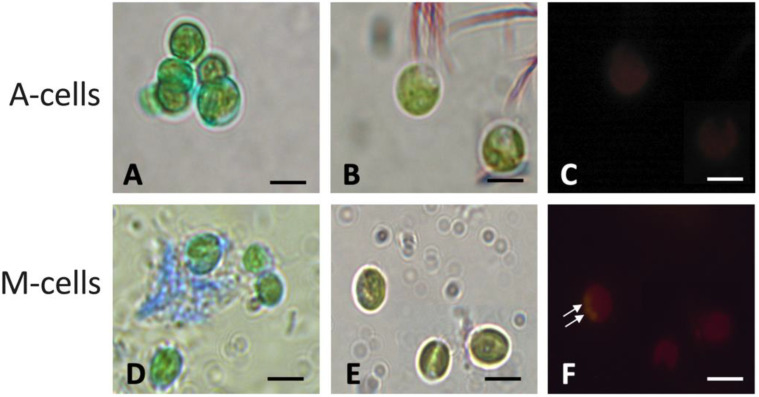
Light microscopy images of *N. oleoabundans* cells belonging to autotrophic (**A**–**C**) and mixotrophic (**D**–**F**) cultures at 28 and 12 d of cultivation, respectively. (**A**,**D**) Pictures taken on cell samples after Alcian Blue staining for exo-polysaccharide (EPS) detection. Blue coloration identifies EPS. (**B**,**C**,**E**,**F**) Pictures taken on cell samples after Nile Red staining for lipid droplets detection. In (**B**,**E**) images of Nile Red-stained cells at the conventional light microscope. In (**C**,**F**), images of the same cells reported in (**B**,**E**) but taken at the epifluorescence microscope (excitation wavelength = 485 nm). In (**C**,**F**), when present, lipid droplets appear as yellowish-gold globules (arrows). A-cells refer to algae in autotrophic cultures, while M-cells refer to algae in mixotrophic ones. Bars: 2.5 μm.

In order to improve information on the biochemical composition of cells, specific staining for acidic exo-mucopolysaccharides (EPS) and for cytoplasmic lipids was performed, and then samples were observed in the light microscope (Figure 5). Observations of microalgae highlighted that cells from culture A were characterized by a limited reaction to Alcian Blue staining at the cell wall level, and very scanty, blue-colored EPS were visible outside cells (Figure 5A). Furthermore, no lipid droplets were visible inside cells, both observing samples using white and fluorescent light lamps (Figure 5B,C). Differently, in culture M, a conspicuous blue staining due to EPS material was visible in the space surrounding the cells, a sign of release from the cells (Figure 5D), and cytoplasmic lipids were observed on NR-stained cells, as highlighted by the presence of yellowish droplets inside some cells; under white light, droplets were less evident (Figure 5E,F). NR staining of M cells confirmed the lipid nature of globules observed at TEM (Figure 4D).

### 2.4. Chemical Characterization of Microalgae Extracts Used for Antiviral Tests

The characterization of extracts showed that extract A, i.e., that obtained using algal biomass from culture A, contained higher amounts (ranging between 2.6 to 3.3×) of all photosynthetic pigments with respect to extract M, i.e. that obtained from culture M cells (Table 1). Moreover, total phenolics were higher in extract A than in extract M, even if the difference was less pronounced (about 24%; 55.2 vs. 42.3 μg_Coum.Ac.Eq._ mL^−1^ in extracts A and M, respectively; *p* < 0.01). Interestingly, total proteins were almost doubled in extract M than in extract A (*p* < 0.001). Due to morphological observations, lipids were assessed only for extract M, where about 1 μg μL^−1^ of those classes of molecules were contained. The GC-MS analysis highlighted the presence of hydrocarbons, fatty acids methyl esters and phytol (Figure S1). For biological activity, the fatty acids profile is of major interest and was assessed. A larger content in saturated than in unsaturated fatty acids was observed (about 68% vs. 32% of total fatty acids). In detail, the saturated palmitic (C16:0) and stearic (C18:0) acids were 54.45% and 13.37%, respectively, while the unsaturated oleic (C18:1) and linoleic (C18:2) fatty acids were, respectively, 11.38 and 20.79% (Table 1).

### 2.5. Antiviral Activity of Microalgae Extracts

**Figure 6:**
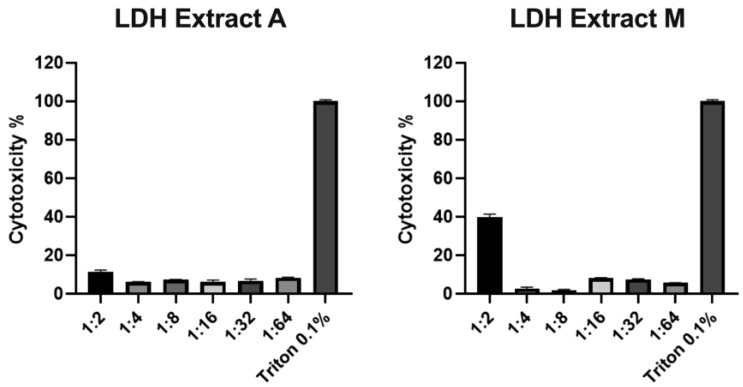
Cell cytotoxicity assay performed by the lactate dehydrogenase (LDH) assay to analyze the effect of different concentrations of *N. oleoabundans* extract A and M, respectively, obtained from autotrophic (A) and mixotrophic (M) algae cultures, after 24 h treatment on human MRC-5 fibroblast cells. Triton 0.1% was used as positive control for 100% cell death. Values are reported as means ± standard deviations (*n* = 3). In each case, *p* value was always below 0.05.

The cytotoxic effect of microalgae extracts A and M on human MRC-5 fibroblast cells was assessed as a preliminary analysis for antiviral activity tests. Both extracts were two-fold diluted in a culture medium (from 1:2 to 1:64). The obtained dilutions were used to treat 24 h cells from the same cell line. After incubation, a lactate dehydrogenase (LDH) assay was performed and showed that extract A was not cytotoxic at all concentrations tested, with no significant difference among concentrations, while extract M was toxic only at the highest concentration tested (1:2, 39.64% cytotoxicity), as shown in Figure 6.

**Figure 7:**
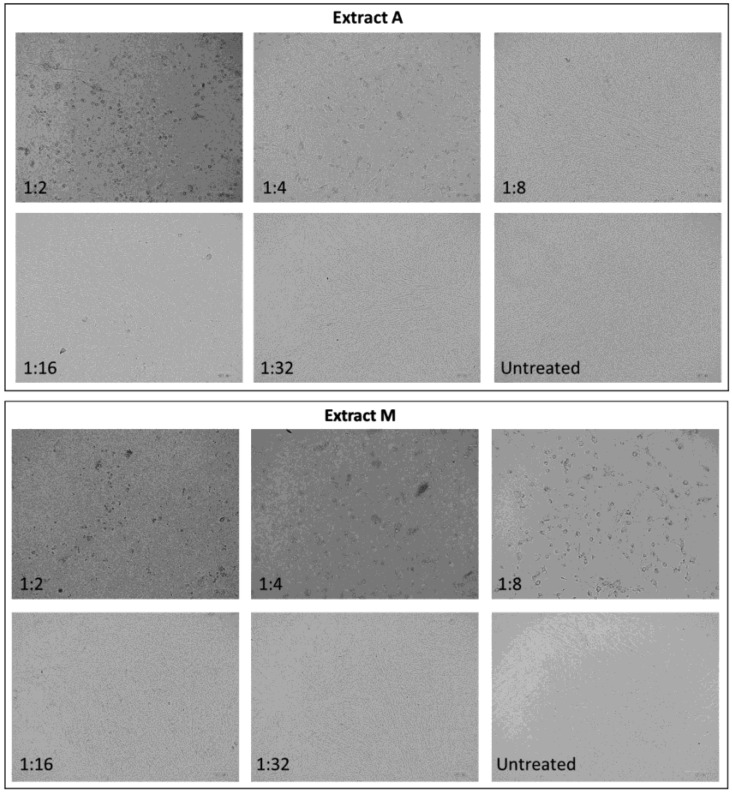
Effects of *N. oleoabundans* extracts A and M, respectively obtained from autotrophic (A) and mixotrophic (M) algae cultures, on human MRC-5 fibroblast cells morphology, after 24 h treatment at different dilutions. The untreated cells exhibited fibroblast morphology that was maintained until dilution of 1:8 and 1:16 for extracts A and M, respectively. MRC-5 treated with large concentration of extracts did not form a complete monolayer and exhibited black nuclei and tense cytoplasm. Cells were observed at ×20 magnification.

Parallel to the LDH test, changes in cell morphology were evaluated by microscopy examination, which highlighted different degrees of alteration in cell shape and detachment from the well surface according to extract and dilutions employed (Figure 7). For this reason, it was chosen to perform the antiviral experiments with the first dilution that did not present morphological alteration, e.g., 1:16 for extract A and 1:32 for extract M, even if cell viability was not affected at higher concentrations.

**Figure 8:**
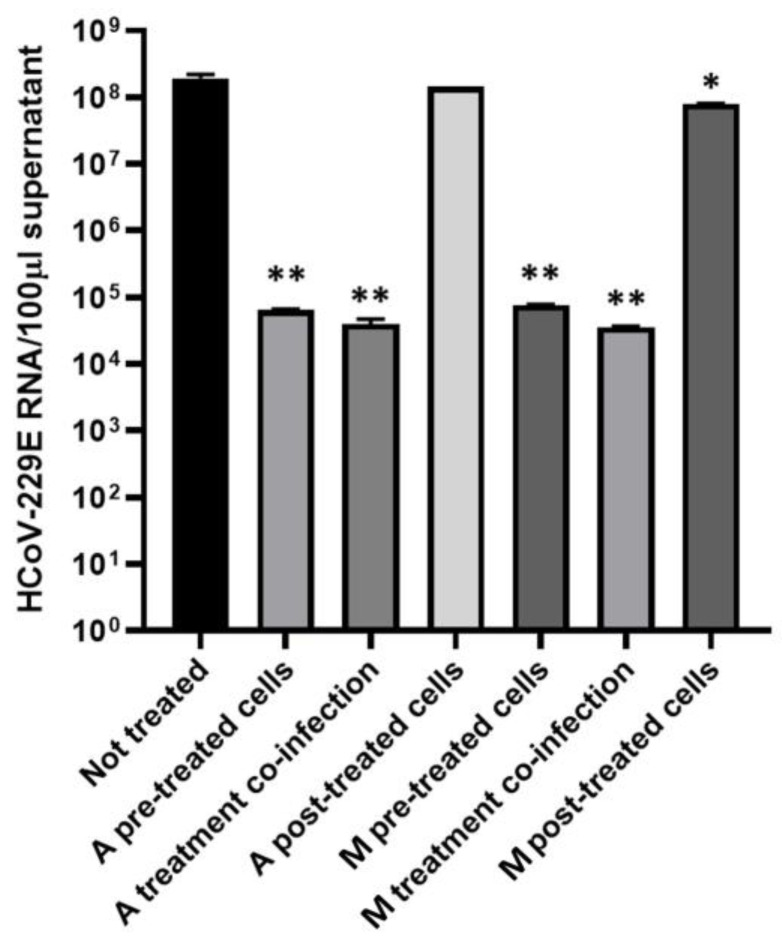
Real-time PCR quantification of HCoV-229E genomes per 100 µL supernatants. Human MRC-5 fibroblast cells were infected with HCoV-229E treated or untreated with *N. oleoabundans* extract A and M at different stages of infection. Extract A derived from autotrophic algae cultures, while extract M from mixotrophic ones. Untreated infected MRC-5 cells were used as control for viral replication. Total RNA was extracted from supernatants collected after 48 h of infection, and viral RNA released in medium was quantified by real-time PCR after reverse transcription. Values are reported as means ± standard deviations (*n* = 3). *, *p* ≤ 0.05; **, *p* ≤ 0.01.

In order to assess the potential antiviral activity of extract A and M, a time-of-drug-addition assay was performed by treating MRC-5 cells at different steps of infection: pretreatment and treatment during infection to evaluate alterations in viral entry and post-infection treatment to evaluate a possible post-entry mechanism (Figure 8). Infected MRC-5 cells without treatment were used as a control of infection. Then, the virus release in the cell medium after 48 h post-infection was examined by quantitation of viral genomes through real-time PCR. It was found that both extracts were able to significantly reduce the viral load when added before or during the infection, with respect to the untreated cells (Figure 8; Student’s *t*-test, *p* = 0.0098). In particular, the logarithmic reduction for extracts A added before or during infection was 3.47 and 3.68, respectively (corresponding to 99.97% and 99.98% of reduction compared to the untreated control). The logarithmic reduction for extract M added before or during infection was 3.40 and 3.73, respectively (corresponding to 99.96% and 99.98% of reduction compared to the untreated control). No significant changes in viral genomes quantity were observed in the post-infection treatment with extract A compared to the untreated; on the contrary, post-infection treatment with extract M was able to reduce the viral load (*p* = 0.0281) significantly, but the logarithmic reduction was only 0.38 (corresponding to 58.05% decrease compared to untreated control).

To clarify if the antiviral activity was exerted during the first steps of infection or possibly by damage to the viral envelope, an infectivity assay was performed. For this purpose, viral inoculum (5.2 × 10^4^ PFU mL^−1^) was treated with extracts for 1 h at room temperature (RT). As a negative control, the untreated virus in the culture medium was put in the same condition. Then MRC-5 cells were infected with a treated or not-treated virus, and a titration by plaque assay was performed. The results showed that both extracts caused a complete drop in HCoV-229E infectivity since no plaque was formed in cells infected with a treated virus, in comparison to the untreated virus (Table 2).

## 3. Discussion

Microalgae are attractive and, even more, are considered crucial as natural sources of bioactive metabolites for developing innovative health compounds [12,17,18]. Indeed, it has been estimated that 9% of natural-sourced biomedical molecules are isolated from algal biomass, and many of them cannot be obtained via synthetic pathways [19,22].

In the present study, two microalgal cultures of *N. oleoabundans* were characterized and used to obtain extracts tested for their antiviral effects against HCoV-229E, here used as a model human *Coronaviridae* virus. Autotrophic and mixotrophic cultures of the alga were employed since it is known that different metabolic pathways are related to modifications of the growth ability and of the morpho-physiological and biochemical features of microalgae [8,10,13,15]. In particular, growth monitoring was intended as a preliminary step to select the proper harvesting time for total extract preparation. Indeed, whatever the end purposes, the criteria of algal productivity must be met for the worthwhile use of microalgae. Differently, morphological observations and biochemical quantifications were intended to highlight molecules with bioactive properties.

For both microalgae samples, biomass for extract preparation was harvested at the early stationary phase of growth (28 or 12 d of cultivation for samples A and M, respectively), when algae reached the highest productivity in terms of cell density and dry biomass yield. A shorter cultivation period and higher growth of algae under mixotrophic conditions with respect to autotrophic ones agree with literature data for the same alga in various media added or not with organic carbon sources [10,15,36,37,38]. Furthermore, at 28 or 12 d of cultivation, cultures A and M were still in an optimal physiological condition, as highlighted by the fluorimetric measurements (F_V_/F_M_ ratio) and by the photosynthetic pigment molar ratios (Chl*a*/Chl*b* and Chls_TOT_/Car). The maximum quantum yield of PSII was always over 0.65, pointing to good “health” conditions of the algae in both cultivations [39,40]. The higher values recorded for the F_V_/F_M_ ratio in the mixotrophic algae samples are very likely referred to lower chlororespiratory processes undertaken when glucose is added to the culture medium, as already demonstrated for *N. oleoabundans* in a mixotrophic brackish medium [10,38,41]. As regards photosynthetic pigments, notwithstanding different concentrations characterized A and M cells during the cultivation period, the molar ratios remained stable, with values around 2 for Chl*a*/Chl*b* and 6–7 for Chls_TOT_/Car. The latter, perfectly in line with fluorimetric results, further suggested that an efficient stoichiometry of photosynthetic pigment was maintained in both cultures [42]; for comparative data on *N. oleoabundans*, see [9,10,26]. In addition to results on biomass yield and the whole physiological status of algae samples, the choice of the harvesting time was further supported by the notion that secondary metabolites, which can exert bioactive properties also as antivirals, are usually produced in larger amounts at the stationary phase of growth when cell duplication is reduced, and the products of the primary metabolism are channeled as substrates for the secondary metabolism [22,43]. In accordance, [22] found that the best antiviral effect against Mayaro Fever (due to the arbovirus MAYV) was obtained using extracts from *A. platensis* at the stationary phase. In those extracts, terpenoids, flavonoids, fatty acids and carotenoids were detected, essentially compounds with antiviral/antioxidant effects [19,22,44]. Similarly, [45] used extracts from several microalgae harvested at the stationary phase of growth for their experiments against hemorrhagic septicemia virus (VHSV) and African swine fever virus (ASFV). Thus, in the present work, a preliminary morphological and biochemical evaluation of some classes of known compounds with antiviral, antioxidant or anti-inflammatory properties has been performed on algae samples only when they reached the stationary phase (i.e., the selected harvesting period for extracts preparation) [19,44]. On the whole, microalgae in culture A reached lower cell density (and gave lower dry biomass yield) than algae in culture M, but they were characterized by higher contents in compounds like pigments, proteins and phenolics. Vice versa, algae from culture M were characterized by higher biomass production, contained intracellular lipids (absent in culture A cells) and released more EPS than culture A. In the mixotrophic sample, active production and release of compounds, very likely entrapped in the EPS matrix, was also supported by the ultrastructural analysis, which showed vesiculation next to the plasma membrane. In this perspective, vesiculations and cytoplasmic material, possibly released in the extracellular surface, are observed in several microalgae, like *Tisochrysis lutea* or *Desmodesmus* sp. [5,46].

All bioactive compounds detected in both *N. oleoabundans* cell samples are generally recognized to be responsible for antiviral actions, and, very recently, a specific exo-polysaccharide fraction of the alga has been demonstrated to play immune-modulatory properties [12]. In that case, the effect on immune function of a selected *N. oleoabundans* EPS fraction, containing glucose, mannose, galactose, xylose, ribose, arabinose and rhamnose, was tested using splenic lymphocytes proliferation and phagocytosis assays [12]. Instead, in the present study, direct tests on HCoV-229E infection on fibroblast cells were performed using whole *N. oleoabundans* extracts. Up to now, studies on the pharmacological properties of *N. oleoabundans* are really at a very early stage, and our work is intended as preliminary research to highlight in vitro antiviral effects of its whole aqueous extracts.

Based on pre-tests of the impact of both extracts on MRC-5 human fibroblast cells viability and morphology, 1:16 dilution was chosen for antiviral tests using extract A, and 1:32 dilution for extract M. Independent of the dose employed, the very interesting result is that extracts from both culture A and M were able to inhibit infection by HCoV-229E. In detail, our trials showed that the administration of the extracts before or during viral infection blocked the replication of the virus, while treatment with extracts on MRC-5 cells after virus infection was not able to block, or at least only reduce, viral replication. Since direct treatment of the virus with extracts completely inhibited infection of fibroblasts, the obvious consequence is that *N. oleoabundans* extracts play their antiviral effects mainly on the first steps of infection by somehow directly altering the virus envelope or making the target cell not suitable for virus attack or uncoating. For example, it is reported that acidic polysaccharides from microalgal EPS play antiviral effects. Acidic polysaccharides contain carboxyl, phosphate or ester groups [19] and have been demonstrated to show activity against Herpes virus simplex (HSV) or vesicular stomatitis virus (VSV) [47,48,49]. This kind of polysaccharide has been morphologically highlighted, mainly in mixotrophic algae. In this sample, also intracellular lipid droplets were observed. In *N. oleoabundans*, likewise in microalgae in general, the composition of these globules is mainly due to triacylglycerols, i.e. neutral lipids, formed by a wide variety of fatty acids [10,50]. Even if fatty acids have only a low affinity for aqueous solvents, extract M showed the presence of palmitic and linoleic acids as the major fractions (about 54 and 21% total FA, respectively), followed by stearic (13%) and oleic acids (11%) (Table 1). Fatty acids often have effects on viruses: among others, in the present study, the action of extract M could be related to the capability of fatty acids to penetrate the surrounding membrane of HCoV-229E, destabilizing its envelope’s architecture [51,52]. However, both extract A and M showed similar effects on HCoV-229E, thus suggesting that not only EPS and lipids from *N. oleoabundans* may have exerted antiviral effects, but also other compounds, or belonging to the classes of compounds we examined, or to other ones. For example, in addition to intracellular lipids, *N. oleoabundans*, as microalgae in general, contains other lipids, for example, those of the photosynthetic membranes (abundant in chloroplasts of both A and M cells), i.e., monogalattosyldiacylgycerols (MGDG), digalattosyldiacylglycerols (DGDG) and sulfoquinovosyldiacylglycerols (SQDG) [53]. This consideration deserves attention since MGDG is a strong viricide of HSV2, even if its mechanism of action is unclear [54], while SQDG, a glycolipid rich in sulfur, has been demonstrated to inhibit DNA-polymerase [55,56]. Thanks to its negative charge, SQDG, which was tested on HIV and HSV viruses, interacts with the positive charge of DNA-polymerase by blocking the bonds with DNA [55,56,57]. Furthermore, both autotrophic and mixotrophic algae obviously contained proteins; they were more abundant in A-cells than in M-ones, even if the total protein content in total extracts used for antiviral tests gave an opposite result (Figure 3A, Table 1). This unexpected result could be linked to different efficiency of cell wall disruption and consequent release of proteins from *N. oleoabundans* cultivated under different growth conditions, as [58] observed for the same alga under fresh or marine N-depleted or -repleted waters. In addition, that finding could be the result of different extractability and solubility of proteins in an aqueous solution with respect to the extraction buffer. Regardless of the different protein concentrations in the two extracts compared to what was found in the starting biomass, it is known that in microalgae, the major proteins are Rubisco and LHCII [9,59], but other proteins are present in addition to these. Among total proteins, lectins are represented in both marine and freshwater microalgae, and have recently been also specifically identified in *N. oleoabundans* [60,61,62]. Interestingly, lectins have significant antiviral properties as preventive agents, which block interactions of viruses (or microbes in general) with the cell membrane since they occupy the sites for virus attachment to the cell membrane [19]. The plausible presence of lectins in the total protein fraction of both extracts supports the effect of inhibiting virus infectivity in the early stages of attachment to the target cell that we observed. In addition, mainly in extract A, the photosynthetic pigment content (chlorophylls and their derivatives—phaeophytin or phaeophorbides—, and carotenoids—in *N. oleoabundans* astaxanthin and lutein are abundant; all these molecules play antiviral effects), together with polyphenols could have contributed to the anti-HCoV-229E activity [28,53,63,64,65,66]. Both these latter two classes of biomolecules are anti-oxidants [17,28,66] and, in some cases, they can directly inhibit plaque formation or inhibit binding of Spike proteins to host-cell receptor ACE2, or also been involved as protease inhibitors, helping to avoid virus entry or stop virus infection (e.g., HIV, SARS, MERS) [19,67,68].

Even if a more specific characterization of algal biomass and of its total extracts has to be set up to identify better and quantify bioactive compounds that they contain and have driven the antiviral effects against HCoV229E (not only those belonging to the chemical classes here evaluated), from present results, it appears that for extract A the antiviral effect may have been mainly promoted by proteins together with pigments and phenolics, present in larger quantities than in extract M. Differently, for extract M the effect may have been mainly due to proteins in association with acidic EPS, and lipids.

## 4. Materials and Methods

### 4.1. Algal Material and Experimental Design

The present research study was performed using the green microalga *Neochloris oleoabundans* UTEX-1185 (Chlorophyta, Sphaeropleales; www.utex.org, accessed on 15 November 2022) cultivated autotrophically (culture A) and mixotrophically (culture M) in BG11 medium (www.utex.org). For mixotrophic cultivations, 2.5 gL^−1^ glucose was added to the medium in accordance with previous papers [10]. In both cases, cultures were maintained in a growth chamber (24 ± 1 °C, 80 μmol_photons_ m^−2^ s^−1^ of photosynthetically active radiation (PAR) with a 16:8 h light-darkness photoperiod) and cultivated with continuous shaking at 110 rpm without CO_2_ addition. Whole extracts were prepared using algal biomass from both cultures and have been employed to test antiviral effects on the human coronavirus HCoV-229E. Cultures A and M were characterized up to the stationary phase of growth (28 d for A and 14 d for M) in order to find the most promising time for the preparation of extracts.

Both microalgal cultures were prepared and processed under sterile conditions. During cultivations, growth of algae was monitored as cell density and dry biomass yield; pH of culture media was monitored as well since it is a parameter linked to growth and photosynthetic activity of algae [69,70]. In addition, a morpho-physiological and biochemical characterization of the algae was carried out. In detail, photosystem II (PSII) maximum quantum yield was monitored during cultivation periods to evaluate the physiological state of algae [71]. In parallel, the content of photosynthetic pigments was measured because of the bioactive and antioxidant properties of this class of compounds. From data on growth and photosynthetic measurements, time 28 (culture A) and 12 d (culture M) were selected for whole extract preparation. At those experimental times, the total protein and the total phenolics contents of algae were spectrophotometrically quantified, while the production of exo-mucopolysaccharides (EPS) and of cytoplasmic lipid droplets was highlighted through morphological observations.

An aqueous whole extract of both algae cultures was prepared (see Section 4.8), characterized in terms of pigments, total protein, total phenolics and lipids (see Section 4.9), and then used to test its effects against the HCoV-229E infection in MRC-5 human fibroblast cells (see Section 4.10 and its subparagraphs).

For all experimentations, tests were set up at least in triplicate.

### 4.2. Growth Evaluations: Cell Density and Dry Biomass of Algae Cultures and pH of Algae Culture Medium

The cell densities (million cells per mL; 10^6^ cells mL^−1^) of cultures A and M were evaluated at the light microscope using a Thoma’s counting chamber (HBG, Giessen, Germany). Cell densities were plotted on a base 2 logarithmic scale to obtain the growth kinetics. On the basis of cell densities, growth rates (µ; d^−1^) were calculated, as well, using the following formula:

µ (d^−1^) = (log_2_ *N*_1_ − log_2_ *N*_0_)/(t_1_ − t_0_) (1)

where µ is the growth rate, *N*_1_ the cell number at time t_1_, *N*_0_ the cell number at time 0 and t_1_ − t_0_ the time interval (days) [72].

Furthermore, dry biomass yield (i.e., grams of algal dry weight, DW, per Liter; g_DW_ L^−1^) of both algal samples were also evaluated, as reported in [10]. In detail, dry biomass weight of algal cultures was evaluated by filtering aliquots of algae samples using pre-weighed and pre-dried glass fiber filters (Whatman GF/C; 1.2 µm pore size); then, filters were rinsed with distilled water, dried for 72 h at 60 °C in an oven and weighted with an Ohaus analytical balance until constant weight was reached [50]. In parallel, the culture medium was harvested by centrifugation (2000× *g*, 10 min) for pH measurements; a Jenway mod. 3510 (Staffordshire, Stone, UK) bench pH-meter was employed.

### 4.3. Evaluation of Algae PSII Maximum Quantum Yield

For the evaluation of the maximum quantum yield of PSII (F_V_/F_M_ ratio), a Junior PAM (Pulse Amplitude Modulation; Heinz Walz GmbH, Effeltrich, Germany) fluorimeter was employed. For the measurements, during cultivation periods, aliquots of algae cultures were harvested by centrifugation at 10,000× *g* for 5 min. The pellet was resuspended in a small aliquot of the supernatant, gently deposited, drop by drop, onto a wet filter paper (Schleicher and Schuell; 1 × 4 cm), and then dark-adapted for 15 min before fluorescence measurements [41]. Basal and maximum fluorescence (F_0_ and F_M_, respectively) values were measured by flashing the samples with a saturating light pulse (0.6 s) and then used for the calculation of the maximum quantum yield of PSII as F_V_/F_M_ ratio, where variable fluorescence F_V_ is F_M_–F_0_ [71].

### 4.4. Photosynthetic Pigment Extraction and Quantification

Algae samples were harvested by centrifugation (8000× *g*, 10 min), and the extraction of photosynthetic pigments was performed with absolute methanol according to [10,72]. The methanolic extracts were manipulated under dim green light to avoid photo-degradation. Optical density of extracts was measured with a Pharmacia Ultrospec 2000 UV-Vis spectrophotometer (1-nm bandwidth; Amersham Biosciences, Piscataway, NJ, USA) at 665 nm (Chlorophyll *a*, Chl*a*), 653 nm (Chlorophyll *b*, Chl*b*), 470 nm (Carotenoids, Car) and 750 nm (background disturbances). Quantification of pigments concentration in the algal biomass, expressed on a DW basis (percentage of pigments over total DW of algae; %DW), was performed using formulae reported in [73] as follows:

Chl*a* (µg mL^−1^) = 15.65 ∗ (A666 − A750) − 7.34 ∗ (A653 − A750) (2)

Chl*b* (µg mL^−1^) = 27.05 ∗ (A653 − A750) − 11.21 ∗ (A666 − A750) (3)

(4) $$\mathrm{Car}\;(µg{\mathrm{mL}}^{-1})=\frac{1000*\left(A470-A750\right)-2.86\;\mathrm{Chl}a-129.2\;\mathrm{Chl}b}{221}$$

where A666, A653, A470 and A750 are the absorbance respectively at 666, 653, 470 and 750 nm.

Results from the formulae were converted into DW% of pigments in the algal biomass by considering the volume and the dry biomass of cultures used for extraction, the total volume of the obtained methanolic extracts, and the dilution factors for spectrophotometric measurements.

Molar ratios of pigments (Chl*a*/Chl*b* and Chls_TOT_/Car) were calculated using the molar concentration of pigments (nmol_PIG_ 10^−6^ cells), still estimated according to formulas above reported, converted into µg_PIG_ 10^−6^ cells, and then divided by the specific molecular weight value of each pigment.

### 4.5. Total Proteins Extraction and Quantification

Total proteins of both algae cultures were extracted and evaluated according to [9,10] with minor modifications. About 50 mL of cultures with an optical density at 750 nm of 1 were harvested by centrifugation (500× *g*, 10 min). The pellet was resuspended in a washing buffer containing 2 mM Na_2_EDTA in 1× PBS buffer and then transferred in Eppendorf tubes for subsequent centrifugation (2000× *g*, 10 min). Thereafter, pellets were resuspended in extraction buffer (0.1 M NaOH, 1% sodium dodecyl sulfate, 0.5% β-mercaptoethanol in distilled water) and kept in liquid N_2_ for 2 min, followed by 2 min in a bath set at 80 °C (3 times). The mixture was then rapidly frozen in liquid N_2_ and left at −20 °C overnight. The day after, pellets were added with glass beads (0.40–0.60 µm diameter; Sartorius, Göttingen, Germany) and vigorously vortexed for 10 min (alternating cycles of 30 s of vortexing with 30 s of cooling in ice). After centrifugation (1500× *g*, 10 min), supernatants were harvested, obtaining an I extract. The pellets were then further extracted using a small aliquot of the same extraction buffer (2 min vortexing, followed by 15 min in a 60 °C bath). After centrifugation, the supernatant (II extract) was added to the first one and used for quantification. The quantitative estimation of proteins in the algal biomass, expressed on DW basis (%DW), was determined with a slightly modified Lowry’s method [74], using bovine serum albumin (BSA) as the standard (Sigma Chemicals, St. Louis, MO, USA).

### 4.6. Total Phenolics Extraction and Quantification

For total phenolics extraction from algae and subsequent quantification, aliquots of algal cultures containing about 120 × 10^6^ cells were harvested by centrifugation (600× *g*, 10 min) and stored at −20 °C until extraction. For the preparation of extracts, algal biomass was mixed with methanol and then exposed to mechanical rupture as follows: 15 min of continuous vortexing, followed by 30 min in an ultrasound bath, and finally, further 10 min-long vortexing ([75], with modifications). Then, samples were centrifugated (1500× *g*, 10 min), and the supernatants were harvested for the quantification with the Folin–Ciocalteu reaction described in [75]. Briefly, 100 µL of samples were left to react for 5 min with 750 µL of Folin–Ciocalteu reagent (Merck, Darmstadt, Germany; 1:10 in distilled water); then, the mixture was added with 750 µL of Na_2_CO_3_ (60 gL^−1^) and let incubate for 1.5 h at RT. The mixtures were measured at 725 nm using the same Pharmacia spectrophotometer described above. The total phenolics concentration of the methanolic extracts was calculated on the basis of a calibration curve prepared using coumaric acid (Sigma Chemicals Co., St. Louis, MO, USA) as the standard and was expressed as mg equivalents of coumaric acid over total DW of algae cultures (mg_Coum.Ac.Eq._ g_DW_^−1^) [76].

### 4.7. Morphological Observations of the Algae

#### 4.7.1. Transmission Electron Microscopy (TEM)

Ultrastructure of algae sample was observed using aliquots of algal samples harvested by centrifugation (600× *g*, 10 min). Cell samples were prepared as follows: for fixation and post-fixation steps, glutaraldehyde (3% *v*/*v* in phosphate buffer 0.1 M, pH 7.2; 3 h, 4 °C) and OsO_4_ (2% *v*/*v* in the same buffer; 1 h, RT) were used, respectively. Then, cells were dehydrated in acetone series and embedded in Araldite resin. Ultrathin sections were finally stained with lead citrate and uranyl acetate [72]. For observations, a Zeiss EM910 (Electron Microscopy Center, University of Ferrara, Ferrara, Italy) was employed.

#### 4.7.2. Light Microscopy

Light microscopy was used to highlight the accumulation of cytoplasmic lipids and the production of cell wall EPS material.

The intracellular presence of lipids was evaluated by staining cells with the fluorochrome Nile Red (NR; 9-diethylamina-5Hbenzo[α]phenoxazine-5-one, 0.5 mg dissolved in 100 mL acetone) (Sigma-Aldrich, Gallarate, Milan, Italy), as described in [26]. Aliquots of 10 µL NR solution were added to 1900 µL of a cell suspension of 1.5 × 10^6^ cells; then the mixture was incubated in darkness at 37 °C for 15 min. After staining, the cells were observed with a Zeiss model Axiophot microscope equipped with an epifluorescence apparatus, using the excitation wavelength of 485 nm (filter set, BP485, LP520); NR dye highlights intracellular lipid droplets as yellowish globules [26].

The production and release of EPS were highlighted by staining cells with Alcian Blue 8GX (Serva) dye, prepared at 1% in 3% acetic acid [77,78]. For the staining, aliquots of 500 μL of sample were harvested by centrifugation (10,000× *g*, 5 min) to separate the algal biomass from the culture medium. Twenty μL of dye was added to the algal pellet; then, the sample was allowed to react for 30 min at RT. After two washes in distilled water to remove excess dye, the algal cells were observed under the conventional light microscope cited above. The blue coloration conferred by the dye indicates the presence of acidic exo-mucopolysaccharides produced at the cell wall level in the algal cells and/or released by the cells [79,80].

In both cases, photographs were taken with a VisiCam Pro 20C digital camera (20 megapixels) mounted on an adaptor.

### 4.8. Preparation of Algal Whole Extracts for Antiviral Tests

According to algae characterization, algal biomasses from both cultures were washed with distilled sterile water and harvested by centrifugation (600× *g*, 10 min), respectively, at times 28 and 12 d of cultivation. Cells were then mechanically disrupted in a cold pre-sterilized mortar in the presence of liquid N_2_ and used to prepare aqueous extracts at a final concentration of 25 g_DW_ L^−1^ using Eagle’s minimal essential medium (MEM), suitable for the cultivation of human MRC-5 fibroblast cells employed to test the antiviral potential of the extracts (see Section 4.10.2). The resulting whole extracts were stocked at 4 °C until analyses.

### 4.9. Chemical Characterization of Extracts Used for Antiviral Tests

Aliquots of whole extracts obtained from both cultures A and M were used to evaluate the contents in photosynthetic pigments (Chl*a* and *b*, and total carotenoids; μg mL^−1^; for calculations, see Formulas (2)–(4) [73]), total proteins (μg μL^−1^), and total phenolics (μg mL^−1^). In detail, aliquots of extracts were diluted in methanol for spectrophotometric quantification of photosynthetic pigments, while total phenolics and total proteins were quantified using the Folin–Ciocalteu reaction [75] and Lowry’s method [74], respectively. Conversely, the fatty acids profile was performed on mixotrophic extracts only due to morphological results. Lipid extraction from algae extract was performed by the Bligh and Dyer method [81], with some modifications. At the algae extract, an equal volume of 1:2 *v*/*v* chloroform/methanol solution was added. After liquid–liquid separation, the chloroform phase was evaporated to dryness; lipids were weighted and referred to as μg over μL of extract (μg μL^−1^); lipids were then recovered in 1 mL of hexane. Fatty acid methyl esters were prepared by transesterification with 5% of sodium hydroxide in methanol solution in order to obtain free fatty acids methyl esters. Sample volumes of 1 μL were injected into the GC-MS apparatus, Varian Saturn 2100 MS/MS, with ion trap mass spectrometer. Separations were performed using a Zebron ZB-WAX Phenomenex capillary column (60 cm in length, 0.25 mm i.d.) supplied with helium carrier gas at 1 mL min^−1^ constant flow. The injector temperature was 250 °C, and the oven temperature program was the following: start 100 °C for 2 min, ramp to 200 °C at 10 °C/min, and hold for 108 min. The peaks were identified based on their retention times using authentic standard fatty acids methyl ester and on their mass spectra compared with NIST library of the instrument. The amount of each fatty acid was expressed relative to the total fatty acid content (semi-quantitation based on area ratio).

### 4.10. Cells and Virus

MRC-5 human fibroblast cells (ATCC CCL171) were grown in monolayers and maintained in Eagle’s MEM with nonessential amino acids (Lonza Biosciences; Lonza, Basel, Switzerland) supplemented with 10% heat-inactivated fetal bovine serum (FBS; Lonza Biosciences) and penicillin (100 units mL^−1^; Sigma-Aldrich), streptomycin (100 μg mL^−1^; Sigma-Aldrich; Italy), L-glutamine (2 mM; Sigma-Aldrich), at 37 °C in 5% CO_2_. Human coronavirus 229E (ATCC^®^ VR-740) was propagated in MRC-5 cells in complete medium with 2% FBS. The supernatant was harvested when cytopathic effect was observed and centrifugated to collect the viral stock. HCoV-229E stock was titrated by plaque assay on MRC-5 cells and stored at −80 °C. For plaque assay, confluent MRC-5 cells were infected with 10-fold serial viral dilutions. The cells were incubated at 33 °C for 1 h and subsequently overlaid with 1.5% methylcellulose (Sigma-Aldrich) to allow plaque formation. The cells were fixed with 70% methanol and stained with 1% crystal violet, and plaques were counted using a stereo microscope (Nikon).

#### 4.10.1. Cell Viability

The effect on cell viability of the two aqueous whole extracts A and M was evaluated on MRC-5 by Cytotoxicity Detection Kit (Lactate DeHydrogenase, LDH) colorimetric assay (Roche), following manufacturer’s instructions. The cytosolic enzyme LDH will be released due to membrane damage into the supernatant and measured spectrophotometrically at 490 nm. Cells were seeded into 96-well plate at a density of approximately 8 × 10^4^ and incubated overnight. The day after, MRC-5 was treated for 24 h with 2-fold serial dilutions (from 1:2 to 1:64) of extracts A and M, and the viability was assessed by LDH assay. Triton-X100 (Sigma-Aldrich) 0.1% was used as positive control for cell death, and untreated cells were used as negative control for viability. The results obtained by LDH assay were expressed as percentage relative to the mean of the positive control, representing 100% cytotoxicity.

#### 4.10.2. Antiviral Activity Test

To assess the antiviral properties of microalgae extracts, the non-toxic dilution of extracts was used. In order to clarify which phase of infection could be potentially affected, 4 conditions were tested: (1) viral inoculum was pretreated with extracts for 1h at RT before infection; (2) cells were pretreated with extracts for 1h before infection; (3) extracts were added during the absorption step, and then removed; (4) cells were treated with extracts after infection. For condition (1), the residual viral infectivity after treatment was assessed by infecting MRC-5 and titrated by plaque assay as described above. For conditions 2, 3 and 4, cell supernatants were collected 48 h post-infection and quantified by real-time PCR after RNA extraction. Briefly, RNAs were extracted with PureLink Viral RNA/DNA mini kit (Thermo Fisher Scientific; Waltham, MA, USA) and reverse-transcribed with High Capacity cDNA reverse transcription Kit (Thermo Fisher Scientific). HCoV-229E quantification was performed by amplification of the ORF1ab gene using the Universal Master Mix (Applied Biosystems; Waltham, MA, USA) as follows: 229E-F: 5′-TTTGATGCTGGAGTCGTAGTG-3′, 229E-R: 5′-ACGGAACACGAAACCCTTAG-3′, 229E-Probe: 5′-FAM-TGGAAGCAAGTGCTGTGTGTCCTA-3′. A standard curve was generated by determination of copy numbers derived from serial dilutions (10^2^–10^8^ copies) of the corresponding gene block (IDT Technologies; Coralville, IA, USA). Copy numbers of the standards were obtained by conversion of nanograms and base pair length of the known reference sequence using Avogadro’s number. Each quantification was run in triplicates.

### 4.11. Statistical Data Treatment

Data were processed with Graphpad Prism 9 (Graph Pad Software, San Diego, CA, USA) and are reported as means ± standard deviations for *n* number of samples. For analyses on cultures characterization, at each experimental time, data were compared using Student’s *t*-test with significance threshold set at *p* < 0.05 (two tails; independent samples). Asterisks in graphs are used to identify the levels of significance: *, *p* ≤ 0.05; **, *p* ≤ 0.01 and ***, *p* ≤ 0.001. In some cases, differences with results obtained at time 0 within the same sample (A or M) are reported in the text. Results obtained from antiviral activity as mean ± standard deviation from triplicate experiments were evaluated for statistical significance by Student’s *t*-test using the same software described above.

## 5. Conclusions

Extracts from autotrophic and mixotrophic cultures of *N. oleoabundans* affect HCoV-229E infectivity, mainly in the early stages of infection, thanks to their complex composition, which still deserves specific investigation. In addition to molecule classes described above, it should be considered that total extracts, prepared using whole micro-organisms just as microalgae, contain other useful compounds, such as vitamins, mineral salts or other ones, which can act in a synergic way to carry out several biotechnologically valuable functions; in the present paper, antiviral effect was evaluated. From a holistic point of view, total extracts could represent a natural arsenal of biomolecules because the rich array of bioactive molecules they contain can be active at different stages of infections or can improve responses by target cells ([20] and references therein). From this perspective, it may be attractive to consider not making selective extractions of bioactive molecules to obtain very promising and green results in the fight against viruses.

## Figures and Tables

**Table 1:** Chemical characterization of *N. oleoabundans* extract A and M, obtained from autotrophic (A) and mixotrophic (M) cultures at 28 and 12 d of cultivation, respectively, and used for antiviral tests. Data refer to means ± standard deviations (*n* ≥ 8 for pigments, phenolics and protein quantification; *n* = 3 for fatty acids profile). Except for lipids, for each compound or class of compounds, asterisks identify significant differences between culture A and M: **, *p* < 0.01; ***, *p* < 0.001.

	Extract A	Extract M
Chl*a* (μg mL^−1^)	215.7 ± 1.69	65.71 ± 9.69 ***
Chl*b* (μg mL^−1^)	97.20 ± 0.33	38.03 ± 3.83 ***
Cars (μg mL^−1^)	14.59 ± 0.21	4.47 ± 2.18 **
Total phenolics (μg_Coum.Ac.Eq._ mL^−1^)	55.21 ± 10.16	42.27 ± 4.95 **
Total proteins (μg μL^−1^)	1.47 ± 0.28	2.86 ± 0.56 ***
Total lipids (μg μL^−1^) Fatty acids composition (% total FA)	-	1.01 ± 0.08
Palmitic acid (16:0)	-	54.45 ± 4.22
Stearic acid (18:0)	-	13.37 ± 1.28
Oleic acid (18:1)	-	11.38 ± 1.65
Linoleico (18:2)	-	20.79 ± 0.70

**Table 2:** Results of plaque assay of treated or untreated HCoV-229E on human MRC-5 fibroblast cells. Treatment of the virus was performed using *N. oleoabundans* extracts obtained from autotrophic (A) or mixotrophic (M) cultures. Count was performed after five days of incubation.

	PFU mL^−1^
Starting inoculum (T = 0)	5.2 × 10^4^
Untreated HCoV-229E (T = 1 h)	3.85 × 10^4^
HCoV-229E + extract A	0
HCoV-229E + extract M	0

## Data Availability

The datasets generated during and/or analyzed during the current study are available from the corresponding author upon reasonable request.

## Supplementary Materials

The following supporting information can be downloaded at: https://www.mdpi.com/article/10.3390/plants12010026/s1, Figure S1: Chromatogram of *N. oleoabundans* algae extract M, derived from mixotrophic cultures. The first part of the chromatogram is referred to as hydrocarbons. Numbered peaks in the chromatogram are: 1 = palmitic acid methyl ester; 2 = stearic acid methyl ester; 3 = oleic acid methyl ester; 4 = linoleic acid methyl ester.
