# Supplementary material for: Characterization of Neochloris oleoabundans under Different Cultivation Modes and First Results on Bioactivity of Its Extracts against HCoV-229E Virus
Source: Plants (Basel). 2022 Dec 21;12(1):26. doi: 10.3390/plants12010026 (PMC9823352; doi:10.3390/plants12010026)

Supplementary material

Figure S1. Chromatogram of *N. oleoabundans* algae extract M, derived from mixotrophic cultures. The first part of the chromatogram is referred to hydrocarbons. Numbered peaks in the chromatogram are: 1 = palmitic acid methyl ester; 2 = stearic acid methyl ester; 3 = oleic acid methyl ester; 4 = linoleic acid methyl ester.

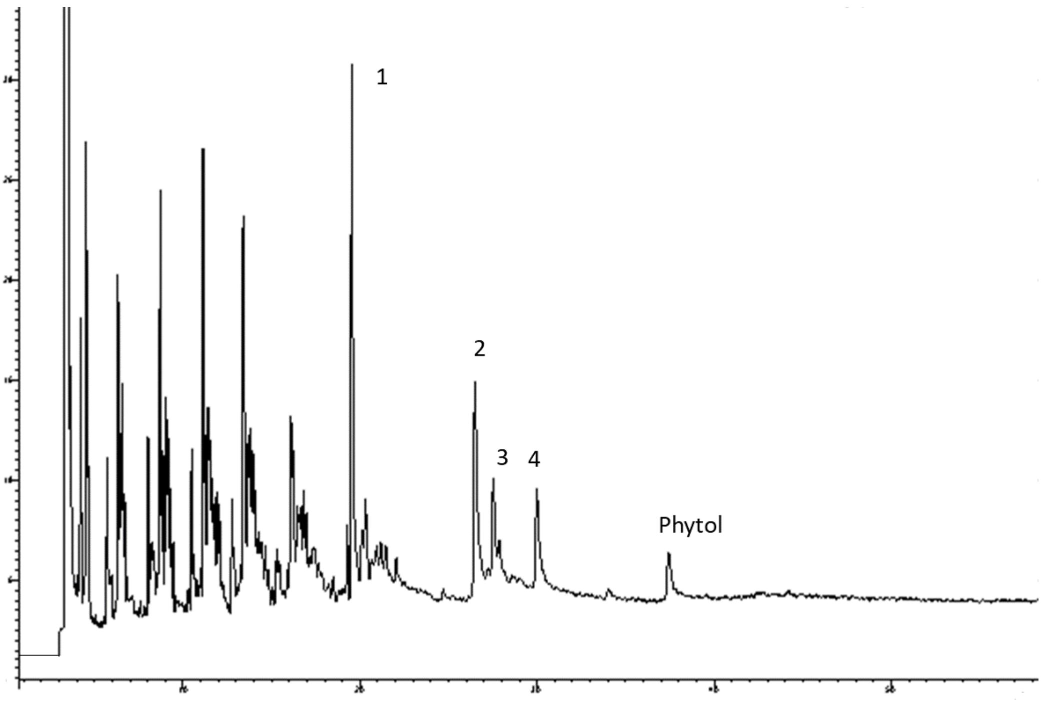

Supplement: Supplementary file 1 [file plants-12-00026-s001.zip › plants-2101932-supplementary.pdf]
